# Effects of “Three Good Things” Intervention on Dyadic Coping Among Young and Middle‐Aged Stroke Survivors and Their Spousal Caregivers

**DOI:** 10.1155/nrp/2745228

**Published:** 2026-08-16

**Authors:** Zhiwei Liu, Xinyue Zhang, Lixia Qu, Yinxia Dou, Lingling Wang, Nan Zhao, Dandan Xiang, Jing Chen, Suyan Chen, Zhenxiang Zhang

**Affiliations:** ^1^ School of Nursing and Health, Zhengzhou University, 101 Science Avenue, Zhengzhou Henan, 450001, China, zzu.edu.cn; ^2^ Department of Traditional Chinese and Western Medicine, The First Affiliated Hospital of Henan Medical University, 88 Jiankang Road, Xinxiang Henan, 453199, China; ^3^ Zhengzhou Health College, No.6 Gongming Road, Zhengzhou Henan, 450064, China; ^4^ School of Nursing, Huazhong University of Science and Technology, 13 Hangkong Road, Wuhan Hubei, 430034, China, hust.edu.cn

**Keywords:** couples, dyadic coping, mutuality, nursing, positive psychology intervention, stroke, subjective well-being

## Abstract

**Aim:**

To evaluate the effectiveness of the “Three Good Things” (TGT) intervention in improving dyadic coping, mutuality, and well‐being among young and middle‐aged stroke survivors and their spousal caregivers.

**Methods:**

A parallel‐group quasiexperimental design was used. One hundred young and middle‐aged stroke survivors and their spousal caregivers were recruited and assigned to either the intervention (*n* = 50) or control group (*n* = 50). The intervention group received eight sessions of the TGT intervention plus routine care. Outcomes were assessed at baseline, immediately postintervention, and at one‐ and three‐month follow‐up. Intervention effects over time and role differences were examined using multilevel modeling.

**Results:**

The intervention group showed significantly higher overall levels of dyadic coping, mutuality, and subjective well‐being than the control group (all *p* < 0.05). Role differences were observed in delegated dyadic coping (*p* < 0.001), with spouses reporting higher levels than patients. Changes over time in delegated dyadic coping differed between patients and spouses (*p* = 0.010), and the intervention effect on mutuality was stronger among patients than among spouses (*p* = 0.001).

**Conclusion:**

The TGT intervention was associated with improvements in psychological and relational outcomes in young and middle‐aged stroke survivors and their spousal caregivers.

## 1. Introduction

Stroke, also referred to as cerebral vascular accident (CVA), is associated with high incidence, recurrence, disability, and mortality rates, as well as a substantial economic burden [1]. In recent years, the incidence of stroke among young (18–44 years) and middle‐aged (45–59 years) individuals has been increasing [1, 2]. Globally, the incidence of ischemic stroke in people aged 18–50 has risen by 40% [3]. Studies show that over two‐thirds of stroke survivors aged 40 and above are between 40 and 64 years old [4]. As stroke often impairs the work capacity of young and middle‐aged individuals and hinders their return to work, it can result in negative emotions and diminished subjective well‐being [5]. Therefore, there is an urgent need to explore novel and effective approaches to improve the subjective well‐being of patients diagnosed with stroke within these age groups.

Most stroke survivors experience multiple disabilities and require long‐term care [6]. Due to a shortage of caregivers, approximately 80% of stroke survivors return home for rehabilitation and rely heavily on their spouses for physical assistance and emotional support [7, 8]. Spousal caregivers bear a significant caregiving burden, leading to increased negative emotions and reduced subjective well‐being [9]. Furthermore, stroke is a traumatic event for both survivors and their spousal caregivers, substantially affecting their individual well‐being and the quality of their relationship [10]. Therefore, it is essential to address the needs of both young and middle‐aged stroke survivors and their spousal caregivers, and to explore effective interventions that enhance their relationship quality and subjective well‐being.

Dyadic coping refers to the joint responses and strategies of couples when facing dyadic stressors [11]. A systematic review by Weitkamp et al. [12] reported that greater dyadic coping among couples affected by chronic illness was associated with better relationship satisfaction and subjective well‐being. However, Song et al. [13] reported that the mean dyadic coping scores were 124.8 ± 27.7 for stroke survivors and 126.7 ± 26.2 for spousal caregivers, indicating moderate levels in both groups. Therefore, it is necessary to explore feasible and accessible interventions to enhance the dyadic coping abilities of stroke survivors and their spousal caregivers.

Various interventions, such as psychological interventions [14], coping skills training [15], and social participation programs [16], have been developed or tested to improve dyadic coping in chronic disease or stroke‐related contexts. However, most available studies have focused on couples facing other chronic diseases or interventions centered primarily on stroke survivors and therefore may not fully address the needs of young and middle‐aged stroke survivors and their spousal caregivers [14–17]. Compared to other chronic conditions, stroke survivors face unique challenges, such as physical impairments, that distinguish them from individuals with other chronic conditions. Young and middle‐aged stroke survivors and their spouses may also face age‐specific pressures, including work disruption, family responsibilities, and caregiving burden. Therefore, there is a need to develop targeted dyadic coping interventions tailored specifically for young and middle‐aged stroke survivors and their spousal caregivers.

Positive psychology is the science of human potential and virtues, which helps individuals discover their inner resources to enhance personal strengths and quality of life [18]. Within positive psychology, the “Three Good Things” (TGT) intervention represents an effective approach for cultivating positive emotions and enhancing well‐being. It involves writing down three things that made one feel happy or content, and reflecting on the reasons behind them, thereby fostering positive emotions [19]. Boiman‐Meshita and Littman‐Ovadia [20] applied the TGT intervention with couples, where both partners recorded three positive experiences and reflected on their causes. The study found that the intervention led to increased happiness and enhanced marital intimacy, without creating a significant time burden on couples. Carrillo et al. [21] conducted a positive psychology intervention among individuals aged 18 to 40 and qualitatively analyzed their recorded entries. The results showed that emotions are a significant predictor of well‐being, and the more positive the emotions expressed in the writings, the greater the improvement in subjective well‐being.

To the best of our knowledge, the TGT intervention has not been applied to young and middle‐aged stroke survivors and their spousal caregivers. Given the unique physical impairments and household burdens faced by young and middle‐aged stroke survivors and their spousal caregivers, which distinguish them from other couples, it is necessary to further investigate the potential of the TGT intervention in improving dyadic coping. Therefore, the purpose of this study is to determine the impact of the TGT intervention on dyadic coping, mutuality, and subjective well‐being among young and middle‐aged stroke survivors and their spousal caregivers.

## 2. Methods

### 2.1. Study Design and Setting

This study adopted a two‐arm parallel quasiexperimental design to evaluate the impact of the TGT intervention on dyadic coping, mutuality, and subjective well‐being among young and middle‐aged stroke survivors and their spousal caregivers. The study was conducted and reported in accordance with the TREND checklist for nonrandomized evaluations of behavioral and public health interventions (Supporting Table S1).

### 2.2. Participants

Participants were recruited from the neurology department of a tertiary hospital in Henan Province between January and September 2022 using a convenience sampling method. Potential candidates were initially identified through a review of electronic medical records by the **first author** based on predefined eligibility criteria. Subsequently, both stroke survivors and their spouses were screened, and their eligibility was confirmed through direct communication. Couples were enrolled only when both partners within the dyad met the inclusion requirements.

The inclusion criteria for stroke survivors were as follows: (1) had a diagnosis of stroke confirmed by CT or MRI; (2) female patients aged 20–59 years or male patients aged 22–59 years, reflecting marriage‐age and age‐group classifications in China; (3) were married and cohabiting with their spouse; (4) demonstrated intact cognitive, verbal, and written communication abilities; (5) possessed a registered WeChat account and were able to use basic messaging functions; and (6) agreed to participate voluntarily. Inclusion criteria for spouses included the following: (1) were legally married to the patient; (2) served as the primary caregiver, providing at least 4 h of care per day; (3) were female aged 20–59 years or male aged 22–59 years; (4) had normal cognitive, verbal, and written communication abilities; (5) had an active WeChat account and were able to use its basic features; (6) provided informed consent.

Patients and spouses were excluded if: (1) both partners could not participate concurrently; (2) either partner had severe comorbidities such as malignancy, heart failure, respiratory failure, or major trauma; and (3) they were enrolled in another research study. Participants were withdrawn if their clinical condition deteriorated or if they chose to discontinue participation.

### 2.3. Sample Size

The sample size was calculated using G∗Power 3.1 software. Based on a two‐way repeated‐measures ANOVA, the required sample size was calculated with a small effect size of 0.15, an *α* level of 0.05, and a power of 0.90, for two groups across four repeated measurements. The calculation indicated that a total sample size of 82 couples was required. Accounting for a predicted 10% attrition rate, the final target sample size was set at 92 couples (46 per group).

### 2.4. Group Allocation and Blinding

Two neurology wards located on different floors within the same hospital were assigned to the intervention and control groups, respectively. To minimize the risk of contamination, nursing and medical staff were fixed within their assigned wards, with no cross‐ward rotations occurring during the study period. Eligible participants from each ward were subsequently invited to join the study. Although blinding of participants, recruiters, and interventionists was not feasible due to practical constraints, data collectors remained blinded to group allocation to minimize detection bias. Because group allocation was conducted at the ward level rather than through individual randomization, this study should be interpreted as a quasiexperimental evaluation.

### 2.5. Intervention Protocol

#### 2.5.1. Intervention Group

The intervention consisted of eight structured sessions delivered over 6 weeks. The initial three sessions were conducted during hospitalization, beginning on the third day after admission. During the first session, stroke survivors and their spousal caregivers received a “Happiness Journal” notebook for recording reflections throughout the intervention. Hospital‐based sessions were conducted either at the bedside or in a private counseling room. Following discharge, the remaining sessions were delivered individually through WeChat video calls. The feasibility of remotely delivered dyadic psychosocial interventions has been supported by prior evidence [17]. Each session lasted approximately 35–40 min and was administered by the **second author** with assistance from the **third author** (see Table 1). All interventionists received a 3‐h standardized training session led by a senior psychological counselor (**the corresponding author**). All patients continued to receive routine clinical care per standard treatment guidelines.

**TABLE 1:** Implementation draft of the “Three Good Things” intervention program for stroke survivors and their spousal caregivers.

Time	Subject	Goal	Contents
1	Introduction	Establish a good interpersonal relationship with stroke couples, assess their psychological state, and explain “Three Good Things.”	1. Introduce the purpose, method, and schedule of the intervention. 2. Assess the dyadic coping, subjective well‐being, and mutuality of stroke survivors and their spousal caregivers. 3. Clearly explain the content and concept of “Three Good Things.” 4. Distribute the “Happiness Journal” notebooks and pens designed for this study and explain how to use them.
2	Experience positive emotions	Enhance the positive coping and happiness of stroke survivors and their spousal caregivers.	1. Recall three things from the week that made the stroke couple happy and are related to each other. 2. Guide the stroke couple to reflect on the reasons behind these events. 3. Guide the stroke couple to write them down in the “Happiness Journal.”
3	Experience positive emotions	Enhance the positive coping and happiness of stroke survivors and their spousal caregivers.	1. Recall three things from the week that made the stroke couple happy and are related to each other. 2. Guide the stroke couple to reflect on the reasons behind these events. 3. Guide the stroke couple to write them down in the “Happiness Journal.”
4	Share positive emotions	Enhance positive coping and mutuality between stroke survivors and their spousal caregivers.	1. Exchange notebooks and read each other’s entries. 2. After sharing, the researcher will guide the stroke couple to discuss the content they recorded.
5	Focus on positive emotions	Enhance positive coping and mutuality between stroke survivors and their spousal caregivers.	1. Recall three things the other person did that made them feel moved or grateful during the week. 2. Guide the stroke couple to write them down in the “Happiness Journal.”
6	Strengthen mutuality	Enhance positive coping and mutuality between stroke survivors and their spousal caregivers.	1. Recall past experiences and guide the stroke couple to write down expressions of gratitude toward each other. 2. Read the written content aloud to each other.
7	Explore inner resources	Enhance positive coping and happiness of stroke survivors and their spousal caregivers.	1. Recall three positive growth experiences of the other person since the onset of the illness. 2. Guide the stroke couple to write these three growth experiences in the “Happiness Journal.”
8	Summarize and envision	Summarize the intervention content and envision a better tomorrow.	1. Guide the stroke couple to summarize the intervention content, exchange notebooks, reflect on the interventions, and encourage the formation of a good habit of continued documentation. 2. Strengthen the stroke couple’s belief in recovery and encourage them with “Tomorrow will be better.”

#### 2.5.2. Control Group

The control group of stroke survivors and their spousal caregivers received the standard health education protocol provided by the neurology ward and were provided with a notebook to record their daily lives without any specific instructions or structured guidance, with no additional activities. Routine health education during hospitalization included an introduction to basic knowledge about the disease and caregiving‐related knowledge, as well as monitoring the patient’s condition, medication guidance, rehabilitation guidance, dietary advice, and psychological care. Postdischarge follow‐up guidance involved checking the patient’s physical condition 1 week after discharge, assessing their status, and providing appropriate guidance. Additionally, health education, medication advice, and dietary guidance were provided to the stroke survivors and their spousal caregivers. One month after discharge, a follow‐up assessment of the patient’s condition was conducted, with corresponding assistance provided based on their needs.

### 2.6. Outcome Measure

#### 2.6.1. Social Demographic and Clinical Data

A researcher‐developed questionnaire collected sociodemographic data from both patients and spouses. Patient variables included age, sex, education, children, per capita income, primary family breadwinner, employment status, and medical insurance type. Clinical data included stroke type, stroke occurrences, complications, and disability. Spousal variables included age, sex, education, employment status, daily care hours, daily caregiving time, and caring for the patient with others.

#### 2.6.2. Dyadic Coping

Dyadic coping was assessed using the Dyadic Coping Inventory (DCI), originally developed by Bodenmann et al. [22] and later adapted into Chinese by Xu et al. [23]. The 37‐item scale comprises six subdimensions: stress communication, supportive dyadic coping, negative dyadic coping, delegated dyadic coping, common dyadic coping, and dyadic coping evaluation, with the dyadic coping evaluation subdimension not included in the total score. Items are scored on a five‐point Likert scale. Negative dyadic coping is reverse‐scored, and the evaluation subscale is not included in the total score. Total scores range from 35 to 175, with higher scores reflecting better dyadic coping. Cronbach’s alpha coefficients in this study were 0.962 for patients and 0.954 for spouses.

#### 2.6.3. Mutuality

Mutuality was measured using the Mutuality Scale [24], which was culturally adapted and translated into Chinese by Shyu et al. [25], with reported evidence of validity and reliability in Chinese populations. This scale consists of 15 items rated from 0 to 4, with total scores ranging from 0 to 60. Higher scores indicate higher levels of mutuality. Cronbach’s alpha values in this study were 0.908 for patients and 0.930 for spouses.

#### 2.6.4. Subjective Well‐Being

Subjective well‐being was assessed using the Index of Well‐being [26], which was culturally adapted into Chinese by Li and Zhao [27]. The scale includes an eight‐item affect scale and a single‐item life satisfaction measure. Scores classify subjective well‐being into low (2.1–6.0), moderate (6.1–10.0), or high (10.1–14.7) levels. Higher scores reflect better well‐being. Cronbach’s alpha values in this study were 0.90 for patients and 0.91 for spouses.

### 2.7. Data Collection Procedures

Data were collected at four time points: baseline (T0), immediately after the intervention (T1), 1 month postintervention (T2), and 3 months postintervention (T3). Questionnaires were collected by the **first author**. Participants completed the questionnaires independently whenever possible. For participants who were unable to complete the questionnaires independently because of educational limitations or stroke‐induced upper limb dysfunction, the researcher assisted by reading the items and recording the responses verbatim. To minimize interviewer bias, the researcher maintained a strictly neutral stance throughout the process. Upon completion, the recorded responses were read back to the participants to verify that the data accurately reflected their true opinions. Completed paper questionnaires were stored securely in a locked cabinet, and electronic data were stored on a password‐protected USB drive. All data entries underwent double‐checking to ensure accuracy.

### 2.8. Data Analysis

Statistical descriptions and group comparisons were performed using SPSS 21.0. Categorical variables were presented as frequencies and percentages. Normally distributed continuous variables were expressed as means and standard deviations (SDs), whereas non‐normally distributed variables were reported as medians and interquartile ranges (IQRs). Group differences were assessed using independent‐sample *t* tests or Mann–Whitney *U* tests, depending on data distribution. Bonferroni corrections were applied to *p*‐values for group comparisons at four time points. Given the repeated‐measures nature of the data, multilevel modeling (MLM) was applied to evaluate changes over time [28]. The MLM analyses were conducted using R 4.6. The performance package was utilized for visual diagnostics of residual normality and homoscedasticity, and the lme4 package was used to construct the models. Role (patient vs. spouse), group (intervention vs. control), and time (T0, T1, T2, T3) were entered as predictors. Interaction terms (time × group, time × role, group × role, time × group × role) evaluated differential trends across roles and groups. A significance level of *p* < 0.05 was applied.

### 2.9. Ethical Considerations

Ethical approval was obtained from the Life Science Ethics Review Committee of Zhengzhou University on November 3, 2020, and the stamped ethics approval document is provided in Supporting Table S2. No formal ethics approval number was assigned by the review committee at that time. The study adhered to the principles of the Declaration of Helsinki. Participants were fully informed of the study aims and procedures and were allowed to withdraw at any time. Written informed consent was obtained from all participants. Additionally, all participants were given access to a copy of the Stroke Health Guide.

## 3. Result

### 3.1. Recruitment and Characteristics of Participants

**FIGURE 1:**
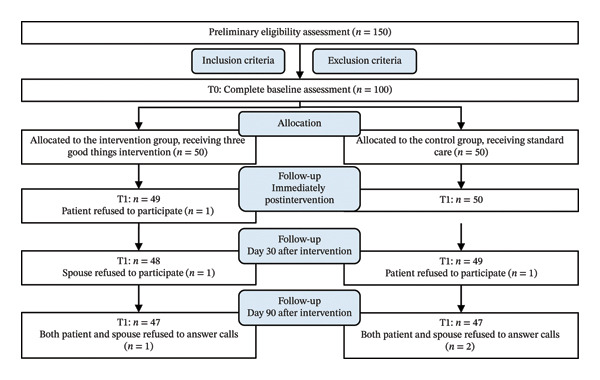
Participant recruitment and intervention flowchart.

During the screening of 150 patient–caregiver dyads, 40 individuals did not meet the eligibility criteria and 10 declined participation. Ultimately, 100 dyads consented and were allocated to either the intervention group (*n* = 50) or the control group (*n* = 50). Both groups had an attrition rate of 6.0%, resulting in 94 dyads being included in the final analysis. Figure 1 illustrates the flow of participant recruitment and outlines the reasons for withdrawal. No significant differences were observed in sociodemographic or clinical characteristics between the two groups included in the analysis.

Table 2 summarizes the sociodemographic and clinical profiles of the 94 dyads. In the control group, patients had a mean age of 52.62 ± 6.78 years, while those in the intervention group had a mean age of 49.87 ± 8.34 years. Most patients were male (67.0%) and had completed senior high school (40.4%). More than half had two or more children (58.5%). The majority were covered by medical insurance for urban residents (70.2%). Ischemic stroke was the predominant diagnosis (73.4%), and over half were experiencing their first stroke event (67.0%). There were no significant differences between the intervention and control groups across baseline patient characteristics, including age, sex, education, stroke type, number of stroke occurrences, complications, and disability score (all *p* > 0.05), indicating good comparability between the groups.

**TABLE 2:** Characteristics of the two groups of patients and their spousal caregivers.

Variables	Patients				Spousal caregivers			
	Control	Intervention	*t/Z/* *χ* ^2^	*p*	Control	Intervention	*t/Z/* *χ* ^2^	*p*
Sex			0.048^a^	0.826			0.048^a^	0.826
Male	31 (66.0)	32 (68.1)			16 (34.0)	15 (31.9)		
Female	16 (34.0)	15 (31.9)			31 (66.0)	32 (68.1)		
Age (mean ± SD)	52.62 ± 6.78	49.87 ± 8.34	−1.893^b^	0.058	52.49 ± 6.84	50.21 ± 8.32	−1.601^b^	0.109
Education			0.158^a^	0.984			1.010^a^	0.818
Primary school or below	8 (17.0)	7 (14.9)			9 (19.1)	8 (17.0)		
Junior high school	15 (31.9)	15 (31.9)			18 (38.3)	18 (38.3)		
Senior high school or technical secondary school	19 (40.4)	19 (40.4)			16 (34.0)	14 (29.8)		
Junior college or above	5 (10.6)	6 (12.8)			4 (8.5)	7 (14.9)		
The number of children			0.555^c^	0.872				
0	2 (4.3)	1 (2.1)						
1	17 (36.2)	19 (40.4)						
2 or above	28 (59.6)	27 (57.4)						
Per capita income			1.300^c^	0.738				
Less than 2000 Yuan	5 (10.6)	8 (17.0)						
2000 to 3000 Yuan	26 (55.3)	22 (46.8)						
3001 to 4000 Yuan	14 (29.8)	14 (29.8)						
4000 Yuan or above	2 (4.3)	3 (6.4)						
Medical insurance			1.369^c^	1.000			2.239^c^	0.358
Medical insurance for urban workers	12 (25.5)	12 (25.5)			8 (17.0)	10 (21.3)		
Medical insurance for urban residents	33 (70.2)	33 (70.2)			38 (80.9)	33 (70.2)		
Commercial insurance	2 (4.3)	1 (2.1)			1 (2.1)	4 (8.5)		
Other	0 (0)	1 (2.1)			0 (0)	0 (0)		
Primary family breadwinner			0.048^a^	0.826				
Yes	32 (68.1)	31 (31.9)						
No	15 (66.0)	16 (34.0)						
Employment status			1.072^a^	0.301			0.043^a^	0.835
On job	19 (40.4)	24 (51.1)			26 (55.3)	27 (57.4)		
Unemployed	28 (59.6)	23 (48.9)			21 (44.7)	20 (42.6)		
Stroke type			1.707^c^	0.569				
Hemorrhagic	11 (23.4)	12 (25.5)						
Ischemic	34 (72.3)	35 (74.5)						
Mixed	2 (4.3)	0 (0)						
Number of stroke occurrences			0.144^c^	1.000				
1	31 (66.0)	32 (68.1)						
2	12 (25.5)	11 (23.4)						
≥ 3	4 (8.5)	4 (8.5)						
Number of complications			2.062^c^	0.646				
0	0 (0)	2 (4.3)						
1	20 (42.6)	20 (42.6)						
2	21 (44.7)	21 (44.7)						
≥ 3	6 (12.7)	4 (8.5)						
Disability^d^			1.535^c^	0.470				
No significant functional disability	35 (74.5)	30 (63.8)						
Mild disability	9 (19.1)	14 (29.8)						
Moderate disability	3 (6.4)	3 (6.4)						
Daily caregiving time (h)							1.713^c^	0.657
≥ 4					0 (0)	2 (4.3)		
≥ 8					11 (23.4)	10 (21.3)		
≥ 12					36 (76.6)	35 (74.5)		
Caring for the patient with others							0.190^a^	0.663
Yes					30 (63.8)	32 (68.1)		
No					17 (36.2)	15 (31.9)		

^a^Pearson chi‐square test, *χ* ^2^ value.

^b^ *Mann–Whitney U* test, *z* value.

^c^ *Fisher–Freeman–Halton* test, *χ* ^2^ value.

^d^Disability was assessed using the Modified Rankin Scale, with higher scores indicating greater disability severity (1 = no significant functional disability, 2 = mild disability, and 3 = moderate disability).

The spousal caregivers had a mean age of 52.49 ± 6.84 years in the control group and 50.21 ± 8.32 years in the intervention group. Most had a junior high school education (38.3%) and were insured under the medical insurance for urban residents (75.5%). Over half provided care for at least 12 h per day (75.5%), and the majority had additional support from another caregiver (66.0%). No significant differences were observed between the intervention and control groups among spousal caregivers in sex, age, education level, daily caregiving duration, and whether they provided care with others (all *p* > 0.05).

### 3.2. Comparison Between the Intervention and Control Groups From T0 to T3

**FIGURE 2:**
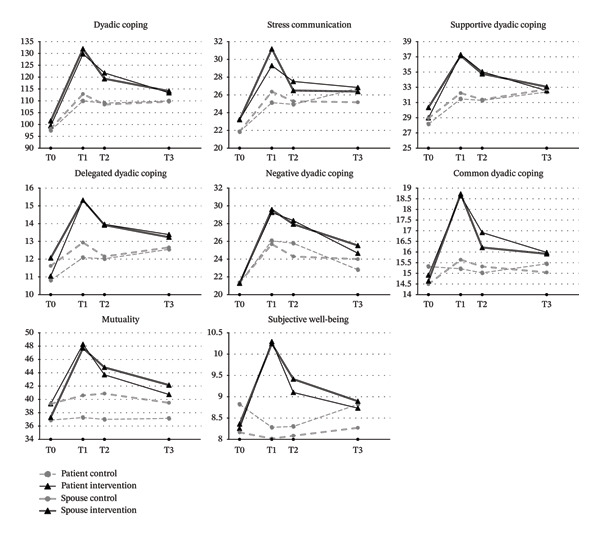
Changes in dyadic coping, mutuality, and well‐being among stroke survivors and their spousal caregivers. Abbreviations: T0, baseline; T1, immediate postintervention; T2, 1 month postintervention; T3, 3 months postintervention.

Table 3 summarizes the baseline data and the results from the three follow‐up assessments for both patients and their spouses. Figure 2 depicts the trajectories of dyadic coping and its subdomains—stress communication, supportive dyadic coping, delegated dyadic coping, negative dyadic coping, and common dyadic coping—as well as changes in mutuality and subjective well‐being across the study period. At baseline, no significant group differences were detected in any of the measured variables.

**TABLE 3:** Comparisons of dyadic coping, mutuality, and subjective well‐being between the intervention and control groups across four time points.

Variable	Time	Control group (mean ± SD)	Intervention group (mean ± SD)	Effect size (Cohen’s *d*)	*Z*/*t*	*p* value	Adj. *p*
*Patients*							
Dyadic coping	T0	97.48 ± 12.81	99.42 ± 10.61	0.16	−0.798^*t*^	0.427	1.000
	T1	109.95 ± 8.75	129.80 ± 11.36	1.96	−7.313^*z*^	**< 0.001**	**< 0.001**
	T2	109.04 ± 8.54	121.76 ± 8.16	1.52	−7.379^*t*^	**< 0.001**	**< 0.001**
	T3	109.93 ± 13.56	113.40 ± 9.83	0.29	−1.643^*z*^	0.100	0.400

Stress communication	T0	21.85 ± 4.57	23.21 ± 3.51	0.33	−1.618^*t*^	0.109	0.436
	T1	25.12 ± 3.24	29.29 ± 4.26	1.10	−4.829^*z*^	**< 0.001**	**< 0.001**
	T2	24.93 ± 3.15	27.51 ± 3.22	0.81	−3.833^*z*^	**< 0.001**	**< 0.001**
	T3	26.74 ± 7.15	26.82 ± 3.66	0.01	−1.041^*z*^	0.298	1.000

Supportive dyadic coping	T0	28.17 ± 5.39	28.97 ± 4.44	0.16	−0.793^*t*^	0.430	1.000
	T1	31.46 ± 3.45	37.29 ± 4.26	1.50	−6.120^*z*^	**< 0.001**	**< 0.001**
	T2	31.27 ± 3.41	35.04 ± 3.60	1.08	−5.201^*t*^	**< 0.001**	**< 0.001**
	T3	32.38 ± 4.96	32.55 ± 3.48	0.04	0.285^*z*^	0.776	1.000

Delegated dyadic coping	T0	10.80 ± 2.30	11.04 ± 2.19	0.11	−0.459^*z*^	0.647	1.000
	T1	12.08 ± 1.54	15.31 ± 2.19	1.71	−6.570^*z*^	**< 0.001**	**< 0.001**
	T2	12.02 ± 1.56	13.95 ± 1.62	1.21	−5.318^*z*^	**< 0.001**	**< 0.001**
	T3	12.55 ± 1.90	13.38 ± 2.04	0.42	−1.920^*z*^	0.055	0.220

Negative dyadic coping	T0	21.34 ± 3.94	21.27 ± 3.44	−0.02	0.084^*t*^	0.934	1.000
	T1	26.06 ± 3.72	29.25 ± 3.55	0.88	−4.249^*t*^	**< 0.001**	**< 0.001**
	T2	25.78 ± 3.70	28.34 ± 2.94	0.77	−3.698^*t*^	**< 0.001**	**< 0.001**
	T3	22.80 ± 3.11	24.65 ± 2.48	0.66	−2.730^*z*^	**0.006**	**0.024**

Common dyadic coping	T0	15.31 ± 3.27	14.91 ± 2.15	−0.14	−0.860^*z*^	0.390	1.000
	T1	15.21 ± 2.50	18.63 ± 3.24	1.18	−4.932^*z*^	**< 0.001**	**< 0.001**
	T2	15.02 ± 2.54	16.91 ± 1.95	0.83	−3.575^*z*^	**< 0.001**	**< 0.001**
	T3	15.45 ± 3.24	15.98 ± 1.95	0.20	−1.250^*z*^	0.211	0.844

Mutuality	T0	2.45 ± 0.59	2.62 ± 0.47	0.32	−1.633^*z*^	0.103	0.412
	T1	2.48 ± 0.57	3.21 ± 0.58	1.27	−5.845^*z*^	**< 0.001**	**< 0.001**
	T2	2.46 ± 0.55	2.91 ± 0.34	0.98	−4.104^*z*^	**< 0.001**	**< 0.001**
	T3	2.47 ± 0.59	2.71 ± 0.36	0.49	−2.515^*z*^	**0.012**	**0.048**

Subjective well‐being	T0	8.82 ± 1.38	8.26 ± 1.51	−0.39	1.895^*t*^	0.061	0.244
	T1	8.28 ± 0.94	10.29 ± 1.73	1.44	−5.243^*z*^	**< 0.001**	**< 0.001**
	T2	8.30 ± 0.95	9.10 ± 1.49	0.64	−2.715^*z*^	**0.007**	**0.028**
	T3	8.81 ± 1.38	8.73 ± 1.57	−0.05	0.293^*t*^	0.770	1.000

*Spouses*							
Dyadic coping	T0	98.23 ± 10.24	101.53 ± 8.14	0.36	−1.728^*t*^	0.087	0.348
	T1	112.82 ± 7.32	131.93 ± 13.48	1.76	−6.883^*z*^	**< 0.001**	**< 0.001**
	T2	108.42 ± 7.90	119.87 ± 8.24	1.42	−5.927^*z*^	**< 0.001**	**< 0.001**
	T3	109.61 ± 10.80	114.12 ± 8.60	0.46	−2.239^*t*^	**0.028**	0.112

Stress communication	T0	21.85 ± 4.57	23.21 ± 3.51	0.33	−1.618^*t*^	0.109	0.436
	T1	26.36 ± 2.47	31.17 ± 7.86	0.83	−6.020^*z*^	**< 0.001**	**< 0.001**
	T2	25.27 ± 3.18	26.48 ± 2.68	0.41	−2.245^*z*^	**0.025**	0.100
	T3	25.17 ± 3.56	26.38 ± 2.65	0.39	−1.893^*z*^	0.058	0.232

Supportive dyadic coping	T0	28.93 ± 4.08	30.34 ± 3.44	0.37	−1.802^*t*^	0.075	0.300
	T1	32.23 ± 2.97	37.14 ± 4.25	1.34	−5.503^*z*^	**< 0.001**	**< 0.001**
	T2	31.38 ± 2.97	34.74 ± 4.22	0.92	−4.060^*z*^	**< 0.001**	**< 0.001**
	T3	32.74 ± 3.77	33.06 ± 3.19	0.09	−0.270^*z*^	0.787	1.000

Delegated dyadic coping	T0	11.61 ± 1.87	12.06 ± 1.90	0.24	−1.147^*t*^	0.254	1.000
	T1	12.93 ± 1.25	15.31 ± 1.99	1.43	−5.840^*z*^	**< 0.001**	**< 0.001**
	T2	12.14 ± 1.23	13.91 ± 1.81	1.14	−5.046^*z*^	**< 0.001**	**< 0.001**
	T3	12.65 ± 1.60	13.23 ± 1.50	0.37	−1.731^*z*^	0.084	0.336

Negative dyadic coping	T0	21.34 ± 3.94	21.27 ± 3.44	−0.02	0.084^*t*^	0.934	1.000
	T1	25.65 ± 3.52	29.57 ± 3.47	1.12	−4.797^*z*^	**< 0.001**	**< 0.001**
	T2	24.29 ± 3.29	27.93 ± 3.18	1.13	−5.057^*z*^	**< 0.001**	**< 0.001**
	T3	24.00 ± 3.04	25.53 ± 2.68	0.53	−2.514^*z*^	**0.012**	**0.048**

Common dyadic coping	T0	14.48 ± 2.69	14.63 ± 2.26	0.06	−0.755^*z*^	0.450	1.000
	T1	15.63 ± 2.09	18.72 ± 2.74	1.27	−5.460^*z*^	**< 0.001**	**< 0.001**
	T2	15.31 ± 2.14	16.21 ± 2.34	0.40	−2.506^*z*^	**0.012**	**0.048**
	T3	15.04 ± 2.76	15.91 ± 1.62	0.38	−1.893^*z*^	0.058	0.232

Mutuality	T0	2.62 ± 0.59	2.48 ± 0.46	−0.26	1.231^*t*^	0.221	0.884
	T1	2.70 ± 0.52	3.18 ± 0.43	1.01	−4.800^*t*^	**< 0.001**	**< 0.001**
	T2	2.72 ± 0.50	2.98 ± 0.35	0.60	−2.929^*t*^	**0.004**	**0.016**
	T3	2.63 ± 0.59	2.80 ± 0.31	0.36	−1.541^*z*^	0.123	0.492

Subjective well‐being	T0	8.39 ± 1.76	8.13 ± 1.21	−0.17	−0.863^*z*^	0.388	1.000
	T1	8.01 ± 1.00	10.24 ± 1.77	1.55	−5.778^*z*^	**< 0.001**	**< 0.001**
	T2	8.08 ± 1.05	9.41 ± 1.52	1.02	−4.112^*z*^	**< 0.001**	**< 0.001**
	T3	8.27 ± 1.51	8.89 ± 1.34	0.43	−1.725^*z*^	0.085	0.340

*Note: z*, Mann–Whitney U test; *t,* independent‐samples *t*‐test; Adj. *p* values were adjusted using Bonferroni correction for four between‐group comparisons across four time points within each variable. T0, baseline; T1, immediate postintervention; T2, 1‐month postintervention; T3, 3 months postintervention. Bold text indicates statistically significant *p* values and Adj. *p* values.

Immediately postintervention, dyads in the intervention group demonstrated significantly better dyadic coping, including higher levels of stress communication, supportive dyadic coping, delegated dyadic coping, negative dyadic coping, and common dyadic coping, as well as greater mutuality and subjective well‐being, compared with the control group (all *Adj.* *p* < 0.001). At the 1‐month follow‐up, all outcomes in the intervention group remained significantly better than those in the control group, except for spouses’ stress communication (*Adj.* *p* = 0.100). At the three‐month follow‐up, patients in the intervention group continued to report significantly higher mutuality (*Adj.* *p* = 0.048), while both patients and spouses showed significantly higher scores in negative dyadic coping compared with the control group (both *Adj.* *p* < 0.05).

### 3.3. Results From the MLM

Adherence to normality and homoscedasticity assumptions for all models was verified via Q–Q plots and scale‐location plots of the residuals. Table 4 presents the outcomes of the MLM analysis illustrating the effects of the intervention on stroke survivors and their spouses. Regarding the main effect of time, significant overall changes were observed in dyadic coping, stress communication, supportive dyadic coping, delegated dyadic coping, and negative dyadic coping (all *p* < 0.05), indicating temporal variation in these outcomes across the four time points. For the main effect of role, delegated dyadic coping showed a significant difference between patients and spouses (*p* < 0.001), indicating that spouses reported higher delegated dyadic coping than patients. For the main effect of group, significant differences were found across all outcome variables (all *p* < 0.05), indicating higher overall levels in the intervention group compared with the control group across the study period. The time × role interaction for delegated dyadic coping was significant (*p* = 0.010), indicating that changes in delegated dyadic coping over time differed between patients and spouses, with patients showing a greater increase over time compared with their spouses. The significant group × role interaction for mutuality (*p* = 0.001) indicated that the intervention effects differed between patients and spouses, with a greater overall improvement observed among patients compared with their spouses across study groups.

**TABLE 4:** Results from the multilevel model.

Variables	Predictor	β	Std_β	SE	df	t	*p*	β (95% CI)	Std_β (95% CI)
Dyadic coping	Role	−0.904	−0.023	0.709	652.000	−1.276	0.202	−2.293, 0.485	−0.082, 0.036
	Group	**5.001**	**0.337**	**0.906**	**236.194**	**5.522**	**< 0.001**	**3.226, 6.776**	0.238, 0.435
	Time	**3.130**	**0.249**	**0.379**	**652.000**	**8.263**	**< 0.001**	**2.388, 3.873**	0.190, 0.308
	Group ∗ Role	−0.064	0.001	0.709	652.000	−0.090	0.928	−1.453, 1.325	−0.058, 0.060
	Time ∗ Role	0.386	0.031	0.379	652.000	1.018	0.309	−0.357, 1.128	−0.028, 0.090
	Time ∗ Group	−0.177	−0.014	0.379	652.000	−0.468	0.640	−0.920, 0.565	−0.073, 0.045
	Time ∗ Group ∗ Role	0.051	0.004	0.379	652.000	0.133	0.894	−0.692, 0.793	−0.055, 0.060

Stress communication	Role	−0.395	−0.005	0.252	652.000	−1.567	0.118	−0.888, 0.099	−0.066, 0.056
	Group	**1.404**	**0.217**	**0.329**	**225.763**	**4.272**	**< 0.001**	**0.760, 2.049**	0.112, 0.323
	Time	**0.931**	**0.216**	**0.135**	**652.000**	**6.917**	**< 0.001**	**0.667, 1.195**	0.154, 0.277
	Group ∗ Role	0.027	−0.005	0.252	652.000	0.106	0.916	−0.467, 0.520	−0.066, 0.056
	Time ∗ Role	0.246	0.057	0.135	652.000	1.829	0.068	−0.018, 0.510	−0.004, 0.118
	Time ∗ Group	−0.237	−0.055	0.135	652.000	−1.758	0.079	−0.501, 0.027	−0.116, 0.006
	Time ∗ Group ∗ Role	−0.035	−0.008	0.135	652.000	−0.257	0.797	−0.299, 0.229	−0.069, 0.053

Supportive dyadic coping	Role	−0.386	−0.046	0.232	652.000	−1.664	0.097	−0.841, 0.069	−0.104, 0.012
	Group	**1.616**	**0.275**	**0.325**	**197.297**	**4.978**	**< 0.001**	**0.980, 2.252**	0.163, 0.386
	Time	**0.932**	**0.223**	**0.124**	**652.000**	**7.514**	**< 0.001**	**0.689, 1.175**	0.164, 0.281
	Group ∗ Role	0.004	0.007	0.232	652.000	0.018	0.985	−0.450, 0.459	−0.051, 0.065
	Time ∗ Role	0.114	0.027	0.124	652.000	0.918	0.359	−0.129, 0.357	−0.031, 0.085
	Time ∗ Group	−0.219	−0.052	0.124	652.000	−1.767	0.078	−0.462, 0.024	−0.110, 0.006
	Time ∗ Group ∗ Role	0.02	0.005	0.124	652.000	0.163	0.871	−0.223, 0.263	−0.053, 0.063

Delegated dyadic coping	Role	**−0.410**	**−0.077**	**0.115**	**652.000**	**−3.558**	**< 0.001**	**−0.635, -0.184**	−0.138, −0.016
	Group	**0.703**	**0.323**	**0.140**	**263.228**	**5.005**	**< 0.001**	**0.428, 0.979**	0.229, 0.417
	Time	**0.382**	**0.193**	**0.062**	**652.000**	**6.208**	**< 0.001**	**0.261, 0.502**	0.132, 0.255
	Group ∗ Role	0.039	0.030	0.115	652.000	0.342	0.732	−0.186, 0.265	−0.031, 0.091
	Time ∗ Role	**0.160**	**0.081**	**0.062**	**652.000**	**2.594**	**0.010**	**0.039, 0.280**	0.020, 0.142
	Time ∗ Group	0.006	0.003	0.062	652.000	0.104	0.917	−0.114, 0.127	−0.058, 0.064
	Time ∗ Group ∗ Role	0.018	0.009	0.062	652.000	0.294	0.769	−0.102, 0.139	−0.052, 0.070

Negative dyadic coping	Role	0.157	−0.002	0.245	652.000	0.639	0.523	−0.324, 0.638	−0.068, 0.065
	Group	**0.672**	**0.239**	**0.263**	**388.465**	**2.557**	**0.011**	**0.157, 1.187**	0.160, 0.317
	Time	**0.777**	**0.201**	**0.131**	**652.000**	**5.922**	**< 0.001**	**0.520, 1.034**	0.134, 0.267
	Group ∗ Role	−0.113	−0.021	0.245	652.000	−0.462	0.645	−0.594, 0.368	−0.088, 0.045
	Time ∗ Role	−0.109	−0.028	0.131	652.000	−0.831	0.406	−0.366, 0.148	−0.095, 0.038
	Time ∗ Group	0.241	0.062	0.131	652.000	1.836	0.067	−0.016, 0.498	−0.004, 0.129
	Time ∗ Group ∗ Role	0.014	0.004	0.131	652.000	0.109	0.913	−0.243, 0.272	−0.063, 0.070

Common dyadic coping	Role	0.129	0.033	0.142	652.000	0.908	0.364	−0.150, 0.408	−0.027, 0.093
	Group	**0.606**	**0.235**	**0.202**	**193.234**	**3.005**	**0.003**	**0.211, 1.001**	0.118, 0.352
	Time	0.108	0.043	0.076	652.000	1.419	0.156	−0.041, 0.257	−0.017, 0.104
	Group ∗ Role	−0.021	0.010	0.142	652.000	−0.146	0.884	−0.300, 0.258	−0.050, 0.070
	Time ∗ Role	−0.025	−0.010	0.076	652.000	−0.329	0.743	−0.174, 0.124	−0.070, 0.050
	Time ∗ Group	0.031	0.013	0.076	652.000	0.412	0.680	−0.118, 0.181	−0.048, 0.073
	Time ∗ Group ∗ Role	0.032	0.013	0.076	652.000	0.426	0.670	−0.117, 0.182	−0.047, 0.073

Mutuality	Role	−0.282	−0.090	0.368	652.000	−0.767	0.443	−1.004, 0.439	−0.142, −0.038
	Group	**1.835**	**0.268**	**0.643**	**146.320**	**2.855**	**0.005**	**0.575, 3.094**	0.132, 0.404
	Time	0.313	0.042	0.197	652.000	1.589	0.112	−0.073, 0.698	−0.010, 0.095
	Group ∗ Role	**1.197**	**0.092**	**0.368**	**652.000**	**3.252**	**0.001**	**0.476, 1.919**	0.039, 0.144
	Time ∗ Role	−0.306	−0.042	0.197	652.000	−1.557	0.120	−0.692, 0.079	−0.094, 0.011
	Time ∗ Group	0.252	0.034	0.197	652.000	1.281	0.201	−0.134, 0.638	−0.018, 0.087
	Time ∗ Group ∗ Role	−0.295	−0.040	0.197	652.000	−1.497	0.135	−0.68, 0.091	−0.092, 0.012

Subjective well‐being	Role	0.106	0.047	0.083	652.000	1.277	0.202	−0.057, 0.27	−0.016, 0.109
	Group	**0.386**	**0.262**	**0.106**	**240.398**	**3.648**	**< 0.001**	**0.178, 0.593**	0.159, 0.365
	Time	0.035	0.025	0.045	652.000	0.796	0.426	−0.052, 0.123	−0.037, 0.088
	Group ∗ Role	−0.132	−0.089	0.083	652.000	−1.587	0.113	−0.296, 0.031	−0.152, −0.026
	Time ∗ Role	−0.023	−0.016	0.045	652.000	−0.506	0.613	−0.110, 0.065	−0.079, 0.047
	Time ∗ Group	0.015	0.011	0.045	652.000	0.337	0.736	−0.072, 0.102	−0.052, 0.074
	Time ∗ Group ∗ Role	−0.004	−0.003	0.045	652.000	−0.095	0.925	−0.092, 0.083	−0.066, 0.060

*Note:* Role: 1, patient; 2, spouse. Group: 1, intervention; 2, control. Time (treated as a continuous variable): T0, baseline; T1, immediate postintervention; T2, 1 month postintervention; T3, 3 months postintervention. Bold text indicates statistically significant *p* values.

## 4. Discussion

To the best of our knowledge, this study is the first to adopt the TGT intervention from positive psychology to enhance the dyadic coping levels of stroke survivors and their spousal caregivers. The findings provided novel insights for the future dissemination and application of the TGT approach among stroke survivors and their spousal caregivers. Notably, the attrition rate for both the intervention and control groups in this study was 6%, which is lower than the attrition rates reported in other dyadic psychological interventions [29]. This lower attrition rate may be attributed to the initial two intervention sessions conducted in the hospital, fostering a strong sense of trust in participants. Furthermore, the participants, being young and middle‐aged, found the online format of out‐of‐hospital interventions more convenient and acceptable. Lastly, the intervention’s focus on written expressions of emotions and mutual feelings between couples aligns with China’s traditional culture of introspection, making it more readily accepted by the participants. Moreover, the participants in this study were young and middle‐aged stroke survivors and their spousal caregivers, who may be more receptive to online interventions. Finally, the TGT intervention encourages emotional expression through writing, involves minimal cognitive load, and is more likely to be accepted by Chinese individuals who are less accustomed to expressing emotions verbally.

Significant group differences were observed in dyadic coping and its dimensions, including stress communication, supportive dyadic coping, delegated dyadic coping, negative dyadic coping, and common dyadic coping, while significant temporal changes were observed in all dimensions except common dyadic coping. This aligns with the results reported by Laird et al. [30] in their research involving people with dementia. A possible explanation is that Chinese individuals tend to be more reserved; thus, encouraging stroke survivors and their spousal caregivers to express their emotions through writing may help improve their communication and enhance mutual support [17, 31]. Moreover, recalling their spouse’s personal growth and moments that moved them can encourage continued collaboration in problem‐solving, strengthen confidence in managing adversity, and thereby enhance the dyadic coping capacity of stroke survivors and their spousal caregivers [32]. The nonsignificant time effect for common dyadic coping suggested that collaborative coping between stroke survivors and their spousal caregivers remained relatively stable over time and may require longer term interaction and adjustment to achieve substantial changes [33, 34]. Although the main effect of role showed that spouses reported higher delegated dyadic coping than patients, the significant time × role interaction in delegated dyadic coping indicated that patients showed greater increases over time than spouses. One possible explanation is that patients had limited physical functioning at baseline and were therefore less able to engage in dyadic stress management, resulting in lower levels of delegated dyadic coping [12]. As patients gradually regained functioning over time, the TGT intervention may have promoted couple communication and collaborative coping, enabling patients to shift from being care recipients to active partners in coping. Consequently, patients became more willing to share their spouses’ stress and responsibilities, leading to greater increases in delegated dyadic coping compared with spouses [12].

Significant group differences were observed in the mutuality of stroke survivors and their spousal caregivers. This may be attributed to the promotion of intimate interactions between patients and their spouses, encouraging both partners to express gratitude and to record moments of happiness shared together, which can enhance their emotional closeness and strengthen their mutuality [35]. Moreover, based on the systemic transactional model, the intervention increased opportunities for stroke survivors and their spouses to express their inner emotions to each other, enabling both partners to feel supported by their spouse [17]. This process fosters mutual benefit within the couple and strengthens their mutuality [11]. Additionally, the significant group × role interaction indicated that the intervention had stronger effects on mutuality among stroke survivors than among their spousal caregivers. From the perspective of the systemic‐transactional model of dyadic coping, the differential effect may reflect differences in how stroke survivors and spousal caregivers experience, communicate, and appraise dyadic coping processes [12, 36]. Stroke survivors may have had greater dependence on spousal support after stroke, making them more responsive to positive emotional exchanges and perceived support during the intervention [12]. In contrast, spousal caregivers often experience sustained role strain and caregiving responsibilities, which may limit their capacity to fully engage with the intervention and weaken improvements in their perceived mutuality [12]. As a result, patients felt more supported and understood, which enhanced their perception of relationship quality [20]. Therefore, by promoting emotional interaction and the expression of positive emotions between stroke survivors and their spouses, mutuality can be improved.

Significant group differences were observed in the subjective well‐being of stroke survivors and their spousal caregivers. This may be because the intervention, guided by positive psychology, encouraged them to experience and share positive emotions, fostering an optimistic mindset. This, in turn, strengthened their inner resilience, increased their attention to positive events in life, and enhanced their subjective well‐being [37]. Spousal caregivers may gain positive experiences from the TGT intervention by recognizing personal growth and value derived from caregiving, which in turn may enhance their subjective well‐being [38]. Furthermore, as mutuality between spouses improves, they are better able to face the challenges posed by illness together, further increasing their subjective well‐being [37]. The nonsignificant time × group interaction suggests that although subjective well‐being differed between groups, the trajectories over time were parallel, indicating a stable intervention effect rather than differential temporal change. A possible explanation is that the intervention effects were not sustained over the long term, suggesting that future research should further explore mechanisms to consolidate and maintain the long‐term effects of the intervention.

### 4.1. Limitations

This study has several limitations. First, participants were recruited from a single tertiary hospital in Henan Province, China, which limits the representativeness of the sample. In addition, to ensure feasibility of implementation, the study included only stroke survivors with mild‐to‐moderate disability, adequate cognitive, verbal, and written communication abilities, and the ability to use WeChat. Therefore, the sample may have been healthier and more functional than the broader stroke survivor population, which may have introduced selection bias and limited the generalizability of the findings. The findings may not be generalizable to stroke survivors with severe disability, cognitive or communication impairment, limited digital access, or those receiving care in other regions or healthcare settings. Future studies should include stroke survivors with varying levels of disability and functional status from diverse geographic and healthcare settings, as well as their spousal caregivers, to enhance the generalizability of the findings.

Second, this study used a quasiexperimental design. The absence of true randomization may introduce potential selection bias, as participants who self‐selected into the study may differ systematically from those who declined participation, which could have had a possible influence on the outcomes. Furthermore, although the two wards were located on different floors of the same hospital with similar environments and staff‐to‐patient ratios, the lack of comparative data may have allowed confounding factors to influence the results. Future research should employ multicenter, large‐sample randomized controlled trials with rigorous randomization procedures to strengthen causal inference and improve generalizability. In addition, pre‐registration of the study protocol and analysis plan is recommended to enhance research transparency.

Third, the three‐month follow‐up period was relatively short, making it difficult to determine the long‐term effects of the intervention. Some effects appeared to attenuate by the three‐month follow‐up, suggesting that the sustainability of the intervention benefits remains uncertain. Future studies should extend follow‐up assessments to 6, 12, and 24 months and examine whether booster sessions are needed to evaluate the sustainability of effects and identify strategies for maintaining intervention benefits.

Fourth, although caregiving time provided by spousal caregivers was comparable between the intervention and control groups, the amount of time stroke survivors spent with their spousal caregivers was not specifically controlled. Therefore, the observed improvements may have been partly attributable to increased interaction time between stroke survivors and their spouses rather than to the specific content of the TGT intervention alone. Furthermore, the additional attention provided by interventionists to the intervention group may have had a potential influence on the outcomes. Future studies should systematically measure couple interaction time and interventionist attention and, where possible, equalize these factors across intervention and control groups to minimize potential confounding effects.

Finally, due to the limited sample size, this study was unable to examine the mediating role of dyadic coping in the relationship between the intervention and outcomes, or the moderating effects of patient and spousal roles. In addition, interventionists received standardized training; however, intervention adherence, session delivery, journal completion, participant engagement, and intervention dose were not systematically documented. This limits the reproducibility of the intervention and makes it difficult to determine which components or level of engagement contributed most to the observed outcomes. Future large‐scale randomized controlled trials are needed to investigate mediation and moderation effects, qualitatively analyze journal content, and examine dose–response relationships between intervention engagement and outcomes. Future studies should also establish a formal fidelity‐monitoring framework, including standardized session checklists, adherence logs, and objective engagement metrics such as session attendance, WeChat session duration, frequency of Happiness Journal completion, and response rates to intervention prompts, to improve reproducibility and strengthen internal validity.

### 4.2. Implications for Clinical Practice

The findings suggest that the TGT intervention may be effective in enhancing positive emotions. Encouraging stroke survivors and their spousal caregivers to record moments of joy and gratitude may help activate positive emotions, thereby promoting dyadic coping, mutuality, and subjective well‐being. Overall, this study provides promising preliminary evidence for positive psychological interventions based on the TGT approach, which warrants further investigation through more rigorous study designs. Importantly, the TGT intervention does not require costly equipment or specialized devices, making it easy to implement and scalable in routine care settings. It is also simple in its implementation and imposes minimal cognitive load, making it well‐suited for individuals who are less comfortable expressing their emotions, particularly within the context of traditional Chinese communication styles, which tend to be more implicit and reserved. Additionally, the finding that stroke survivors benefit more than their spousal caregivers underscores the importance of tailoring interventions to the distinct needs of different family members. These features support its potential for wider application across diverse populations.

## 5. Conclusions

This study provides promising preliminary evidence that the TGT intervention may improve dyadic coping, mutuality, and subjective well‐being among stroke survivors and their spousal caregivers. The findings provide preliminary support for the potential value of positive psychological interventions in fostering positive coping and enhancing well‐being. While the current study reports outcomes up to 3 months postintervention among stroke survivors and their spousal caregivers, future research should include longer follow‐up periods and more diverse populations to further examine the potential effectiveness of the intervention in promoting positive emotions and well‐being.

## Author Contributions

Zhiwei Liu: methodology, formal analysis, data curation, and writing–original draft. Xinyue Zhang, Lixia Qu, and Yinxia Dou: investigation and writing–review and editing. Lingling Wang and Nan Zhao: investigation. Dandan Xiang: writing–review and editing. Jing Chen: visualization. Suyan Chen: conceptualization, supervision, and visualization. Zhenxiang Zhang: conceptualization, supervision, project administration, and funding acquisition.

## Funding

This work was supported by the National Natural Science Foundation of China (Grant No. 72174184), the Innovation Scientists and Technicians Troop Construction Projects of Henan Province (Grant No. 134200510018), Key Laboratory of Geriatric Long‐term Care (Naval Medical University), Ministry of Education (Grant No. LNYBPY‐2025‐09), and Key Specialized Research and Development Breakthrough in Henan Province (Grant No. 242102310021).

## Ethics Statement

This study received approval from the Zhengzhou University Life Sciences Ethics Committee on November 3, 2020.

## Conflicts of Interest

The authors declare no conflicts of interest.

## Supporting Information

Additional supporting information can be found online in the Supporting Information section.

## Supporting information


**Supporting Information** Supporting Table S1. TREND statement checklist indicating where each reporting item is addressed in the manuscript. Supporting Table S2. Zhengzhou University Life Sciences Ethics Review Committee Ethics Review Report.

## Data Availability

The data that support the findings of this study are available from the corresponding author upon reasonable request.
