# Supplementary material for: Effects of “Three Good Things” Intervention on Dyadic Coping Among Young and Middle‐Aged Stroke Survivors and Their Spousal Caregivers
Source: Nurs Res Pract. 2026 Aug 16;2026:2745228. doi: 10.1155/nrp/2745228 (PMC13478729; doi:10.1155/nrp/2745228)
Supplement: Supplementary file 1 [file NRP-2026-2745228-s001.zip › Supplementary Table S2.pdf]

Zhengzhou University Life Sciences Ethics Review Committee

Ethics Review Report

**Project Title:** A Study on the Dyadic Coping and Tertiary Responses of Stroke Patients and Their Spouses Based on an Emotion Response Interaction Model	
**Principal Investigator:** Zhenxiang Zhang	
**Ethics Review Opinion:**<br>After review by the Ethics Review Committee of Zhengzhou University Life Sciences, the committee has determined that the content and procedures of this study comply with the ethical requirements for biomedical research issued by relevant international and national authorities. The committee agrees that this project may be submitted for the 2020 Scientific Research Program of the Chinese Nursing Association.<br><br>Zhengzhou University Life Sciences Ethics Review Committee<br>November 3, 2020	

郑州大学生命科学伦理审查委员会

伦 理 审 查 报 告

**项目名称：**	基于发展-情境应对模型的脑卒中患者夫妻二元应对轨迹及干预研究
**项目负责人：**	张振香

伦理审查意见：

经郑州大学生命科学伦理审查委员会审查，该项目研究内容和过程遵循国际及国家颁布的有关生物医学研究的伦理要求，同意该项目申请中华护理学会 2020 年度科研课题。

郑州大学生命科学伦理审查委员会

2020 年 11 月 03 日
